# Universal Use of Novel Oral Anticoagulant Prophylaxis in Myeloma Patients Undergoing IMiD-Based Therapy: Real-World Experience

**DOI:** 10.3390/jcm15020453

**Published:** 2026-01-07

**Authors:** Yasa Gul Mutlu, Ebrar Uzunabdullah, Duha Yahya, Hasan Basri Ergün, Süreyya Yiğit Kaya, Senem Maral, Hüseyin Saffet Beköz, Leylagül Kaynar, Ömür Gökmen Sevindik

**Affiliations:** 1 Department of Hematology, Gaziantep City Hospital, Gaziantep 27470, Türkiye; 2 Faculty of Medicine, Istanbul Medipol University, Istanbul 34214, Türkiye; ebrar.uzunabdullah@outlook.com (E.U.); doha.abdelwahed@std.medipol.edu.tr (D.Y.); hbergun@gmail.com (H.B.E.); 3 Department of Hematology, Istanbul Medipol University, Istanbul 34214, Türkiye; sureyya.yigitkaya@medipol.com.tr (S.Y.K.); senem.maral@medipol.com.tr (S.M.); huseyin.bekoz@medipol.com.tr (H.S.B.); leylagul.kaynar@medipol.com.tr (L.K.); 4 Department of Hematology, Istanbul Florence Nightingale Hospital, Istanbul 34381, Türkiye; gokmen.sevindik@florence.com.tr

**Keywords:** multiple myeloma, thromboprophylaxis, novel oral anticoagulants

## Abstract

**Background/Objectives**: Multiple myeloma (MM) patients receiving immunomodulatory drugs (IMiDs) are at increased risk of venous thromboembolism (VTE). Standard prophylaxis typically involves aspirin or low-molecular-weight heparin (LMWH), guided by risk assessment tools such as SAVED and IMPEDE-VTE. However, these models have practical limitations, and real-world evidence supporting novel oral anticoagulants (NOACs) as primary prophylaxis remains limited. **Methods**: In this retrospective, single-center study, we analyzed 101 MM patients treated with IMiD-based therapy between January 2020 and December 2024. All patients received NOAC prophylaxis (apixaban 2.5 mg twice daily or rivaroxaban 10–20 mg once daily), irrespective of baseline thrombotic risk. Clinical characteristics, comorbidities, and treatment details were collected. The primary outcome was objectively confirmed VTE, while secondary outcomes included bleeding events and treatment feasibility, assessed by treatment continuation without clinically significant bleeding. **Results**: Median age was 63 years (range 35–89); 36.6% were female. Lenalidomide and pomalidomide were used in 86.1% and 13.9%, respectively. Twenty-eight patients (27.7%) had relapsed/refractory disease, while 72.3% were newly diagnosed. Over a median NOAC exposure of 6 months, two patients (2.0%) developed VTE (both deep vein thrombosis). One major bleeding event (1.0%) occurred. **Conclusions**: Universal NOAC prophylaxis in MM patients receiving IMiD-based therapy was associated with a low incidence of thromboembolic events and an acceptable safety profile. These real-world findings suggest that NOACs may represent a practical and effective alternative to aspirin or LMWH, potentially overcoming the limitations of score-based prophylaxis strategies.

## 1. Introduction

Multiple myeloma (MM) accounts for approximately 10% of all hematologic malignancies and is associated with a substantially increased risk of thromboembolism through diverse pathogenetic mechanisms, including disease-related factors, treatment modalities, and patient-specific comorbidities [1,2,3]. In the general cancer population, the incidence of venous thromboembolism (VTE) exceeds 7%; however, among hematologic malignancies, patients with MM carry the highest risk of thrombosis [4].

Early clinical trials of immunomodulatory drugs (IMiDs) without pharmacologic prophylaxis reported VTE rates as high as 4.1 per 100 patient-months, highlighting the need for preventive strategies. As a result, current guidelines recommend prophylactic use of low-dose aspirin or low-molecular-weight heparin (LMWH) in patients receiving IMiD-based therapy, guided by individual thrombotic risk assessment [5]. Nevertheless, there is no clear consensus on the optimal risk stratification tool, with several scoring systems—such as SAVED, IMPEDE-VTE, and PRISM—being proposed but not universally adopted [6,7,8]. Moreover, concerns remain regarding the limited efficacy of aspirin in high-risk patients and the compliance and quality-of-life challenges associated with daily LMWH injections.

Novel oral anticoagulants (NOACs) have become established agents for thromboprophylaxis in patients with solid tumors and non-malignant conditions [9]. Their predictable pharmacokinetics, fixed oral dosing, absence of routine monitoring requirements, and favorable safety profile make them an attractive option in the myeloma setting. While emerging data suggest that NOACs may provide effective and safe prophylaxis in patients treated with IMiDs, real-world evidence remains scarce [10,11,12].

Given these considerations, our study sought to evaluate the feasibility, safety, and clinical outcomes of a simplified, universal prophylaxis strategy in which all MM patients receiving IMiD-based therapy were treated with NOACs, irrespective of baseline risk score classification. This real-world single-center analysis aims to provide meaningful insight into VTE incidence, bleeding complications, and the overall practicality of NOAC use in routine clinical practice, thereby contributing valuable evidence to ongoing discussions regarding optimal prophylaxis approaches in MM.

## 2. Materials and Methods

### 2.1. Study Design and Patient Population

This retrospective single-center study included adult patients diagnosed with multiple myeloma (MM) who underwent IMiD-based induction therapy and received prophylactic c novel oral anticoagulants (NOACs) during the treatment period. Patients who were followed up between January 2020 and December 2024 were retrospectively identified from institutional records. Eligible patients received either lenalidomide or pomalidomide as part of induction or relapse therapy, in monotherapy or combination regimens. Patients on lenalidomide maintenance at 10 mg/day without dexamethasone were excluded from the study. Patient records were reviewed to collect demographic characteristics (age, sex, body mass index), disease status at inclusion (newly diagnosed vs. relapsed/refractory), treatment regimens, and type and dose of IMiD administered. The use of concomitant dexamethasone, daratumumab, carfilzomib, or other agents was also recorded. Details of NOAC prophylaxis—including the type of NOAC (apixaban 2.5 mg twice daily or rivaroxaban 10 mg once daily), start and stop dates, and duration of therapy—were retrieved. Clinical risk factors for thrombosis, such as prior history of venous thromboembolism (VTE), cardiovascular comorbidities (diabetes mellitus, hypertension, cardiovascular disease), body mass index, epoetin use, and peri-treatment surgical interventions, were documented. In addition to routinely collected clinical variables, laboratory parameters relevant to thrombotic risk—including renal function tests and baseline hematologic indices—were reviewed when available. Data were abstracted from electronic medical records using a standardized data collection form to ensure consistency and minimize information bias.

### 2.2. Outcomes and Definitions

The primary outcome was the incidence of objectively confirmed venous thromboembolism (VTE), defined as deep vein thrombosis or pulmonary embolism occurring during IMiD therapy under NOAC prophylaxis. Secondary outcomes included bleeding complications, treatment discontinuation, and overall adherence to NOAC therapy. Disease status at the time of thrombotic events (remission vs. active disease) was also evaluated. VTE events were required to be confirmed by appropriate imaging modalities, including Doppler ultrasonography for suspected deep vein thrombosis and computed tomography pulmonary angiography for suspected pulmonary embolism. Bleeding complications were classified as major or minor according to the criteria of the International Society on Thrombosis and Haemostasis (ISTH). Major bleeding was defined as bleeding associated with hemodynamic compromise, a decrease in hemoglobin level of ≥2 g/dL, need for transfusion, or bleeding in a critical anatomical site. Minor bleeding events included clinically relevant non-major bleeding episodes such as epistaxis or mucocutaneous bleeding that did not require intervention. Chronic kidney disease (CKD) was defined as an estimated glomerular filtration rate below 60 mL/min/1.73 m^2^ persisting for at least three months. Overweight and obesity were defined according to body mass index (BMI), with overweight defined as a BMI of 25.0–29.9 kg/m^2^ and obesity as a BMI ≥ 30.0 kg/m^2^.

### 2.3. Follow-Up and Treatment Exposure

Patients were followed from the initiation of concurrent IMiD and NOAC therapy until discontinuation of either treatment, disease progression, death, or the last available follow-up visit. Duration of NOAC exposure was calculated based on documented start and stop dates. Treatment adherence and discontinuation were assessed through clinical documentation and prescription records.

### 2.4. Statistical Analysis

Descriptive analyses were performed to summarize patient characteristics and treatment-related variables. Patients were categorized according to the presence or absence of venous thromboembolism (VTE), and baseline clinical features—including age, disease status, IMiD type, dexamethasone exposure, obesity, diabetes mellitus, hypertension, coronary artery disease, chronic kidney disease, prior VTE, erythropoietin use, and recent surgery—were descriptively compared between groups. Categorical variables were reported as frequencies and percentages, and continuous variables were expressed as medians with ranges. Given the very low number of thromboembolic events, no formal hypothesis testing or inferential statistical comparisons were performed, and no *p*-values are reported. All analyses were conducted using standard statistical software (SPSS Version 22).

### 2.5. Ethical Considerations

The study was conducted in accordance with the principles of the Declaration of Helsinki and was approved by the local institutional ethics committee. Due to the retrospective nature of the study, the requirement for informed consent was waived. All patient data were anonymized prior to analysis to ensure confidentiality.

## 3. Results

### 3.1. Patient Demographics, Clinical Characteristics, and Treatment Exposure

A total of 101 patients were included in the analysis. The median age at initiation of IMiD therapy was 63 years (range, 35–89), and 37 patients (36.6%) were female. Most patients were treated in the newly diagnosed setting (73 patients, 72.3%), while 28 patients (27.7%) received IMiD-based therapy for relapsed disease. Lenalidomide was the most frequently administered IMiD (87 patients, 86.1%), followed by pomalidomide (14 patients, 13.9%).

IMiDs were administered in combination with dexamethasone in 77 patients (76.2%), whereas 24 patients (23.8%) received IMiD therapy without corticosteroids. Among patients exposed to dexamethasone, 57 (56.4%) received a cumulative dose of 160 mg per month, while 44 (43.6%) received lower doses. At baseline, 16 patients (15.8%) were overweight and 19 (18.8%) were obese. Comorbid conditions were common, including diabetes mellitus (28.7%), hypertension (43.6%), and ischemic heart disease (23.8%). Chronic kidney disease (CKD) was present in nine patients (8.9%).

Twelve patients (11.9%) underwent surgical procedures during IMiD therapy, most commonly kyphoplasty or vertebroplasty. Erythropoietin or darbepoetin was used in 10 patients (9.9%) for anemia management. A prior history of thrombosis was documented in three patients (3.0%). Baseline demographic, clinical, and treatment characteristics are summarized in Table 1.

### 3.2. NOAC Prophylaxis Characteristics and Treatment Duration

For thromboprophylaxis, rivaroxaban was administered to 82 patients (81.2%), while 19 patients (18.8%) received apixaban. Rivaroxaban dosing varied according to clinical judgment and comorbidities: 58 patients (57.4%) received 10 mg daily, 18 (17.8%) received 15 mg daily, and 6 (5.9%) received 20 mg daily. The median duration of concurrent IMiD and NOAC exposure was 6 months (range, 2–38).

Overall adherence to NOAC prophylaxis was high. NOAC therapy was continued without interruption in 100 patients (99.0%), and only one patient (1.0%) permanently discontinued anticoagulation due to an adverse event. Detailed anticoagulation exposure and treatment outcomes are presented in Table 2.

### 3.3. Thromboembolic Events

Thromboembolic events were observed in two patients (2.0%) during IMiD therapy with NOAC prophylaxis. One patient developed recurrent acute deep vein thrombosis of the left popliteal vein, and the other experienced acute femoral vein thrombosis. Both events occurred during rivaroxaban prophylaxis at a dose of 10 mg daily and were managed by escalation to therapeutic dosing (20 mg daily), resulting in complete clinical resolution. Pulmonary embolism was excluded in both cases by computed tomography pulmonary angiography.

Both thromboembolic events occurred in patients treated in the newly diagnosed setting. No VTE events were observed among patients receiving IMiD therapy for relapsed disease or among those treated with pomalidomide.

### 3.4. Bleeding Complications and Safety Outcomes

Bleeding events were infrequent. One patient (1.0%) experienced grade ≥3 lower gastrointestinal bleeding while receiving rivaroxaban 10 mg once daily, secondary to a refractory gastrointestinal plasmacytoma, requiring local intervention and administration of activated prothrombin complex concentrate (aPCC). In this case, NOAC therapy was permanently discontinued, and the patient was switched to a non-IMiD–based regimen (Kd-PACE).

Two additional patients (2.0%) developed minor epistaxis; one occurred in a patient receiving rivaroxaban 10 mg once daily and the other in a patient receiving apixaban 2.5 mg twice daily, who did not require treatment modification. No fatal bleeding events were observed. Importantly, patients receiving higher rivaroxaban doses (15–20 mg daily) did not demonstrate an increased bleeding risk.

### 3.5. Risk Factors Associated with Venous Thromboembolism

Baseline demographic, disease-related, and treatment-related variables were descriptively evaluated according to the occurrence of venous thromboembolism (VTE). No clear differences were observed with respect to age, sex, disease status, IMiD type, dexamethasone exposure, obesity, diabetes mellitus, hypertension, ischemic heart disease, erythropoietin use, or peri-treatment surgical history. A detailed descriptive comparison of VTE-positive and VTE-negative patients is provided in Table 3.

Although diabetes mellitus and hypertension were numerically more frequent among patients who developed VTE, these observations should be interpreted cautiously due to the very small number of events. Notably, both patients who experienced VTE had relevant clinical comorbidities, including chronic kidney disease and/or a prior history of thrombosis, highlighting these factors as potential contributors to residual thromboembolic risk despite NOAC prophylaxis.

## 4. Discussion

In this retrospective real-world study, we evaluated the safety and effectiveness of universal NOAC prophylaxis in multiple myeloma patients receiving IMiD-based therapy. The incidence of venous thromboembolism was low (2%), and major bleeding events were rare (1%), despite the inclusion of patients with multiple comorbidities and recognized thrombotic risk factors. These findings suggest that routine NOAC prophylaxis may represent a feasible and well-tolerated strategy for thromboprophylaxis in IMiD-treated myeloma patients in everyday clinical practice.

Optimal thromboprophylaxis strategy remains a major clinical challenge in myeloma patients receiving IMIDs [5]. A distinctive feature of our study is the universal use of NOAC prophylaxis, independent of baseline thrombotic risk scores such as SAVED or IMPEDE-VTE. Current guidelines advocate for a risk-adapted approach, yet the practical application of these models is often difficult. Risk scores are inherently dynamic, as patients’ clinical characteristics—such as comorbidities, renal function, treatment regimen, or performance status—may change over time, leading to shifts in estimated risk. Moreover, these scoring systems do not capture all clinically relevant variables, and their implementation can vary widely across institutions.

By adopting a universal prophylaxis strategy, our intent was to simplify decision-making and ensure that all patients, regardless of baseline risk, received an effective and convenient preventive approach. While this may theoretically expose some low-risk patients to overtreatment, our data suggest that the safety profile of NOACs is favorable, supporting the feasibility of a streamlined prophylaxis model in routine practice [13].

Although both the SAVED and IMPEDE-VTE scores were developed and validated in similar clinical settings, they differ in design and variable weighting. The SAVED model was derived specifically from cohorts of IMiD-treated MM patients, whereas IMPEDE-VTE incorporated a broader patient population and a distinct methodological framework. Consequently, each captures thrombotic risk from a slightly different perspective, reflecting the multifactorial pathogenesis of VTE in MM. Nevertheless, substantial overlap exists, and neither tool comprehensively integrates the dynamic clinical features that influence risk over time. These limitations further underscore the challenges of relying solely on static risk models and provide additional rationale for simplified, universal prophylaxis strategies, such as routine NOAC use.

From a clinical perspective, our findings have important practical implications. Universal NOAC prophylaxis offers a simplified and standardized approach that may reduce variability in clinical decision-making and minimize the risk of under-prophylaxis, particularly in busy real-world settings. The oral administration, fixed dosing, and favorable safety profile of NOACs may further enhance treatment adherence and patient quality of life compared with parenteral anticoagulants. In this context, routine NOAC use may represent an attractive alternative to complex risk-adapted strategies, especially in patients receiving combination regimens associated with dynamic and evolving thrombotic risk.

In this real-world analysis of 101 multiple myeloma patients receiving IMID-based therapy with NOAC prophylaxis, the incidence of venous thromboembolism was remarkably low (2%), with only one major bleeding event (1%) observed in the context of a gastrointestinal plasmacytoma. These findings suggest that NOACs may represent a safe and effective thromboprophylaxis strategy in patients receiving IMiD-based therapy, although this approach requires validation in larger prospective studies [14]. Both thromboembolic events occurred in patients with multiple comorbidities, including diabetes and hypertension, and one had additional coronary artery disease and chronic kidney disease. Both thromboembolic events occurred in patients with multiple comorbidities, including diabetes and hypertension, and one had additional coronary artery disease and chronic kidney disease. Notably, chronic kidney disease and a prior history of thrombosis were present among patients who developed VTE, findings that are consistent with recognized clinical risk factors incorporated in established models such as SAVED and IMPEDE-VTE. Although other variables, including diabetes and hypertension, were numerically more frequent among patients with VTE, these observations should be interpreted cautiously, given the very limited number of events. Importantly, despite the presence of high-risk clinical features in a subset of patients, the overall VTE incidence remained low under universal NOAC prophylaxis. The safety profile of NOAC prophylaxis in our cohort was also reassuring. Only one major bleeding event (1%) was observed, which occurred in the unique clinical context of a bleeding gastrointestinal plasmacytoma rather than drug toxicity per se. Minor bleeding episodes, such as self-limited epistaxis, were rare and did not necessitate treatment discontinuation. Overall, this supports the reliability of NOACs as a well-tolerated option for thromboprophylaxis in IMID-treated myeloma patients, particularly when compared with the inconvenience and reduced quality of life associated with long-term LMWH injections.

Several prospective and observational studies have evaluated the role of direct oral anticoagulants for thromboprophylaxis in patients with multiple myeloma receiving IMiD-based therapy. Cornell et al. reported favorable safety and efficacy outcomes with apixaban prophylaxis, demonstrating very low rates of venous thromboembolism and no major bleeding events in a prospective cohort [15]. Similarly, Sayar et al. observed low thromboembolic event rates with apixaban in IMiD-treated myeloma patients, further supporting the feasibility of DOAC use in this setting [16]. In addition, Piedra et al. compared rivaroxaban with aspirin in newly diagnosed multiple myeloma patients receiving IMiD-based regimens and reported lower thromboembolic rates with rivaroxaban, with an acceptable bleeding profile [17].

Collectively, these findings are further supported by recent meta-analytic evidence, which demonstrates consistently low rates of venous thromboembolism and major bleeding across studies evaluating DOAC prophylaxis in multiple myeloma [18]. While most available data derive from relatively small prospective cohorts or well-defined observational studies, the overall consistency of outcomes strengthens the rationale for DOAC use in this population. In this context, our real-world analysis complements existing evidence by reflecting routine clinical practice and by adopting a universal prophylaxis strategy independent of formal risk stratification, with similarly low rates of thromboembolic and bleeding events.

Our results contribute to the growing evidence base supporting NOACs as a feasible thromboprophylaxis option in IMiD-treated MM patients. Prior studies have underscored several practical advantages of NOACs—namely, oral administration, predictable pharmacokinetics, fixed dosing, and absence of routine monitoring—which collectively enhance patient adherence and satisfaction. While large randomized data remain limited, multiple retrospective and early-phase prospective analyses have consistently reported low rates of thromboembolism and bleeding with apixaban and rivaroxaban in this context. Notably, in clinical trials evaluating aspirin or low-molecular-weight heparin prophylaxis in IMiD-treated multiple myeloma patients, venous thromboembolism rates have generally been reported in the range of approximately 5–10% with aspirin and 3–6% with LMWH, with major bleeding rates typically below 1–2%. In this context, our real-world cohort demonstrated a low incidence of venous thromboembolism (2%) and major bleeding (1%) under NOAC prophylaxis, while underscoring the need for further prospective evaluation [19,20].

Several limitations of this study should be acknowledged. The retrospective and single-center design may limit the generalizability of our findings, and the relatively small number of thromboembolic events precluded multivariable analysis. In addition, thrombotic risk scores such as SAVED or IMPEDE-VTE were not prospectively applied, reflecting real-world practice but limiting formal comparisons with risk-adapted strategies. Nevertheless, this cohort provides valuable real-world evidence supporting the safety and feasibility of universal NOAC prophylaxis. Prospective, multicenter studies are warranted to further validate this approach and to define the optimal thromboprophylaxis strategy in IMiD-treated multiple myeloma patients.

## 5. Conclusions

Taken together, these findings support the feasibility of NOACs as a safe and effective thromboprophylaxis strategy in patients with multiple myeloma receiving IMiD-based therapy, while also highlighting the challenges of exploring potential risk factors for VTE in real-world cohorts with a limited number of events. In this real-world cohort, universal NOAC prophylaxis was associated with a low incidence of venous thromboembolism and a favorable safety profile, with minimal major bleeding events. The simplicity of oral administration, high treatment adherence, and avoidance of parenteral anticoagulation may offer meaningful advantages in routine clinical practice, particularly in a patient population characterized by dynamic thrombotic risk and prolonged treatment exposure. While prospective randomized studies are warranted to further define optimal patient selection and dosing strategies, our results suggest that routine NOAC prophylaxis represents a pragmatic and well-tolerated approach to thromboprophylaxis in IMiD-treated multiple myeloma.

## Figures and Tables

**Table 1:** Baseline demographic, clinical, and treatment characteristics of patients with multiple myeloma receiving IMiD-based therapy and NOAC prophylaxis.

*n* = 101		*n* = 101
Age (median) years		63 (35–89%)
Gender	Female	37 (36.6%)
	Male	64 (63.4%)
Disease status	Newly diagnosed	73 (72.3%)
	Relapsed	28 (27.7%)
IMID Received	Lenalidomide	87 (86.1%)
	Pomalidomide	14 (13.9%)
IMID Alone/with Dexamethasone	Alone	24 (23.8%)
	With Dexamethasone	77 (76.2%)
Dexamethasone dose per month	160 mg/month	57 (56.4%)
	<160 mg month	44 (43.6%)
BMI	Overweight	16 (15.8%)
	Obese	19 (18.8%)
	Normal	66 (65.4%)
DM	Yes	29 (28.7%)
	No	72 (71.3%)
Hypertension	Yes	44 (43.6%)
	No	57 (56.4%)
Ischemic heart disease	Yes	24 (23.8%)
	No	77 (76.2%)
Surgery	Yes	12 (11.9%)
	No	89 (88.1%)
EPO/Darbepoetin use	Yes	10 (9.9%)
	No	91 (90.1%)
Chronic Kidney Disease	Yes	9 (8.9%)
	No	92 (91.1%)
History of thrombosis	Yes	3 (3%)
	No	98 (97%)

Data are presented as numbers (%) unless otherwise specified. Age is reported as median (range). NOAC: novel oral anticoagulant; BMI: body mass index; DM: diabetes mellitus; CKD: chronic kidney disease; EPO: erythropoietin.

**Table 2:** Anticoagulation characteristics, thromboembolic events, bleeding complications, and treatment continuation during IMiD-based therapy.

*n* = 101		*n* = 101
NOAC	Rivaroxaban	82 (81.2%)
	Apixaban	19 (18.8%)
Median duration of IMID/NOAC exposure months		6 (2–38%)
Thromboembolic events	Deep vein thrombosis (popliteal)	1 (1%)
	Femoral vein thrombosis	1 (1%)
	No Thrombo-embolic event	99 (98%)
Bleeding events	Grade ≥ 3 GI bleeding	1 (1%)
	Minor epistaxis	2 (2%)
	No bleeding event	98 (97%)
NOAC discontinuation due to adverse events	Discontinued because of adverse event	1 (1%)
	Continued	100 (99%)

Data are presented as numbers (%) unless otherwise specified. Duration of IMiD/NOAC exposure is reported as median (range). NOAC: novel oral anticoagulant; IMiD: immunomodulatory drug; GI: gastrointestinal.

**Table 3:** Clinical characteristics and risk factors associated with venous thromboembolism.

*n* = 101		VTE+	VTE−
Number of patients		2	99
Disease status	Newly diagnosed	2	81
	Relaps refractory	0	20
IMID	Lenalidomide	2	85
	Pomalidomide	0	14
Dexamethasone exposure	160 mg/month	1	56
	<160 mg/month	1	43
Overweight/Obese	Yes	1	34
	No	1	65
DM	Yes	2	27
	No	0	72
HT	Yes	2	42
	No	0	57
CAD	Yes	1	23
	No	1	76
CKD	Yes	1	8
	No	1	91
History of VTE	Yes	1	2
	No	1	97
Erythropoietin use	Yes	1	1
	No	10	91
History of surgery	Yes	1	1
	No	11	88

Data are presented as the number of patients unless otherwise specified. VTE: venous thromboembolism; IMiD: immunomodulatory drug; DM: diabetes mellitus; HT: hypertension; CAD: coronary artery disease; CKD: chronic kidney disease.

## Data Availability

The data supporting the findings of this study are available from the corresponding author upon reasonable request.
